# Percutaneous Closure of Aortic-Pulmonary Artery Fistula in Cardiogenic Shock: The Modified Tootsie Roll Technique

**DOI:** 10.1016/j.jaccas.2025.104684

**Published:** 2025-08-20

**Authors:** Billal Mohmand, Faraj Kargoli, Mustafa Suppah, Abeeha Naqvi, Michael Morris, Orazio Amabile, Philip A. Gideon, Marvin H. Eng

**Affiliations:** a Division of Cardiology, Banner University Medical Center, University of Arizona Phoenix, Phoenix, Arizona, USA; b Division of Radiology, Banner University Medical Center, University of Arizona Phoenix, Phoenix, Arizona, USA; c Division of Cardiothoracic Surgery, Banner University Medical Center, University of Arizona Phoenix, Phoenix, Arizona, USA; d Department of Medicine, Creighton University Phoenix, Phoenix, Arizona, USA; e Department of Cardiology, Creighton University Phoenix, Phoenix, Arizona, USA

**Keywords:** acute heart failure, aorta, complication, echocardiography, hemodynamics, imaging, left-sided catheterization, occluder, pulmonary circulation, right-sided catheterization

## Abstract

**Background:**

Aorto-pulmonary fistulas, often resulting from prior aortic surgeries, can lead to severe cardiac complications and are traditionally managed surgically.

**Case Summary:**

This case involves a 62-year-old man with a history of aortic stenosis and multiple surgeries, who presented with decompensated heart failure due to a fistula from a dehiscent aortic tube graft to the pulmonary artery, causing severe right ventricular failure. Given the high surgical risk, a novel percutaneous approach using a modified “Tootsie Roll technique” was employed, involving the deployment of a VBX-covered stent and a ventricular septal defect Amplatzer occluder, which significantly improved hemodynamics and resolved cardiogenic shock.

**Discussion:**

This case underscores the potential of catheter-based techniques as viable alternatives or complements to surgery for managing complex aorto-pulmonary fistulas, particularly in high-risk patients, and highlights the essential role of comprehensive imaging in guiding such interventions, despite the absence of specific guidelines in current American College of Cardiology/American Heart Association recommendations.

## History of Presentation

A 62-year-old male with a complex cardiac history, including a prior Ross procedure and subsequent mechanical aortic valve replacement, presented with progressive dyspnea, fatigue, and lower-extremity edema over several days. On arrival, he was found to be in cardiogenic shock with signs of severe right ventricular (RV) failure, prompting immediate admission to the cardiac intensive care unit.

**Take-Home Messages**

• The “modified Tootsie Roll technique” offers a novel, effective percutaneous solution for closing aortic-pulmonary artery fistulas in high-risk surgical patients.
• Multimodal imaging, like cardiac magnetic resonance imaging and transesophageal echocardiography, is crucial for accurate diagnosis, procedural planning, and real-time guidance in complex cardiovascular cases.
• Transcatheter-based interventions offer viable alternatives for high-surgical-risk patients, emphasizing the need for innovative, individualized treatments when guidelines are limited.

## Physical Examination

The patient appeared acutely ill with hypotension (blood pressure: 85/55 mm Hg), tachycardia (heart rate: 110 bpm), jugular venous distention, and significant bilateral lower-extremity edema. Auscultation revealed a holo-systolic murmur consistent with severe tricuspid regurgitation and findings suggestive of pulmonary regurgitation. Pulmonary auscultation demonstrated bilateral rales.

## Past Medical History

The patient had a significant cardiac surgical history beginning with a Ross procedure 28 years prior to treat severe aortic stenosis. This was later complicated by aortic autograft regurgitation, necessitating mechanical aortic valve replacement. He subsequently underwent surgical repair of an ascending aortic aneurysm. His medical background was further complicated by stage 3 chronic kidney disease, long-standing essential hypertension, and conservatively managed peripheral artery disease. These comorbidities contributed to his overall high surgical risk and complex clinical presentation.

## Differential Diagnosis

• Aortic-pulmonary artery fistula: most likely given his surgical history and clinical presentation.
• Mechanical aortic valve failure complicated by valve regurgitation or thrombus: considered but ruled out based on multimodality imaging including echocardiography and magnetic resonance imaging (MRI).
• Pulmonary embolism: less likely, given the lack of supporting imaging or clinical evidence.
• Acute myocardial infarction: considered but excluded based on negative biomarkers and electrocardiography.

## Investigations

Right- and left-heart catheterization revealed severe tricuspid and pulmonary homograft regurgitation. Transesophageal echocardiography (TEE) demonstrated aortic tube graft dehiscence with pseudoaneurysm formation and fistulous communication with the pulmonary artery (Video 1). Cardiac MRI confirmed the 10 × 15 mm aortic-pulmonary artery fistula with severe RV failure due to pulmonary regurgitation (Video 2).

## Management

Given the patient's elevated surgical risk due to prior sternotomies, renal impairment, and hemodynamic instability, a transcatheter approach was deemed the most appropriate. Using ultrasound, fluoroscopic, and angiographic guidance, a 6-F sheath was placed and upsized to a 16-F Gore DrySeal sheath after deploying 2 ProGlide closure devices. Following full anticoagulation with intravenous heparin, a pigtail catheter was positioned in the aortic root for an aortogram to visualize the fistula (Video 3).

A pulmonary artery catheter was used to access the left main pulmonary artery, and the aortic defect was engaged using a JL3.5 guide and crossed with a glidewire, which was then snared and externalized to create a rail. Attempts were made to cross the defect with an EBU3.75 guide and fine cross catheter. Despite multiple attempts, delivery of a septal occluder device via the antegrade approach was unsuccessful, and the procedure was deferred for possible placement of a pulmonary artery covered stent.

After re-evaluation and multidisciplinary discussions, the decision was made to re-attempt aortopulmonary (AP) fistula closure using a modified “tootsie roll” technique, utilizing the VBX stent as a rail to guide the occluder through the fistula tract. The second attempt was initiated with the placement of a 16-F sheath into the left femoral artery. A pigtail catheter inserted into the aortic root confirmed the site and size of the fistulous connection via aortography. The pulmonary artery was accessed using a Judkins Right 4 catheter, and a guidewire was advanced from the aortic side, captured from the pulmonary side, and externalized to establish a stable rail. Using balloon sizing, the defect was measured and prepared for device deployment (Video 4). A VBX 11 mm × 29 mm covered stent was delivered retrograde across the fistula to facilitate guiding the occluder through the fistula. To ensure closure and reduce the chance of persistent shunting, a 10-mm Amplatzer ventricular septal defect occluder was deployed within the stent from the pulmonary side (Video 5). This dual-device closure strategy—termed the “modified Tootsie Roll technique” which has been used previously for different purposes^1^—ensured optimal apposition and minimized the risk of leak or migration. Postdeployment imaging with angiography (Video 6) and TEE (Video 7) confirmed near-complete resolution of the shunt and improved right-sided pressures.

## Discussion

This case raises 2 central clinical questions: 1) Can catheter-based techniques effectively replace or emerge as a bridge for surgery in managing acquired AP artery fistulas? 2) What is the role of comprehensive imaging in guiding these interventions?

Addressing the first, although surgery remains the standard for AP fistulas, the increasing use of transcatheter approaches is reshaping treatment paradigms, particularly for patients deemed inoperable. Previous reports illustrate successful outcomes using occluder devices or stents, especially in congenital or postsurgical fistulas.^2^, ^3^, ^4^ More recently, Oikonomou et al.^5^ reported on endovascular repair of thoracic aortic pathology, including fistulas, emphasizing minimal invasiveness and rapid stabilization. Similarly, Tarantini et al.^6^ described using Amplatzer devices in thoracic pseudoaneurysm and fistula settings with favorable hemodynamic outcomes.

Our case extends these findings by combining both device types in a hybrid fashion, supporting the utility of this strategy for wide-neck or irregular defects. The modified “Tootsie Roll technique”—to our knowledge—has not yet been documented in this precise anatomical context, marking this report a contribution to the growing evidence base for alternative closure options.

On the second question, advanced imaging proved indispensable from diagnosis to follow-up. Cardiac MRI helped delineate the extent of the fistula, while TEE enabled real-time procedural monitoring. These tools allowed for accurate localization, optimal sizing, and successful deployment without complications. As highlighted by contemporary literature, multimodal imaging not only aids in diagnosis but also reduces procedural uncertainty and improves outcomes in structural heart disease interventions.^1^, ^2^, ^3^, ^4^ This reinforces the growing reliance on imaging-based decision-making in complex cardiovascular procedures.

While the 2020 American College of Cardiology/American Heart Association Guidelines for the Management of Patients with Valvular Heart Disease provide detailed recommendations on aortic regurgitation, tricuspid valve disease, and prosthetic valve management, they do not explicitly address acquired AP fistulas.^7^

This omission highlights a current gap in formalized guidance for similar conditions. Our case emphasizes the importance of individualized, multidisciplinary approaches when encountering such uncommon but clinically significant lesions—which can offer effective solutions when conventional surgery is unfeasible.

## Follow-Up

Postprocedure, the patient stabilized hemodynamically and was weaned off inotropes within 48 hours. RV function improved significantly, and the patient was discharged with cardiology follow-up and plans for serial imaging to monitor device integrity and RV function.

## Conclusions

The modified Tootsie Roll technique offers a safe and effective solution for the closure of acquired aortic-pulmonary artery fistulas in patients unsuitable for surgery. Through the integration of novel device deployment strategies and precision imaging, this case expands the paradigm of percutaneous structural interventions.

## Funding Support and Author Disclosures

Dr Morris has relationships with HeartFlow, Inc and Medtronic. Dr Eng is a Clinical Proctor for Edwards Lifesciences and Medtronic. All other authors have reported that they have no relationships relevant to the contents of this paper to disclose.

## Appendix

Video 1 TEE Image Showing Fistula

Video 2 Cardiac MRI Showing 10 × 15-mm Fistula

Video 3 Preclosure Aortogram

Video 4 Aortopulmonary Fistula Rail Formation

Video 5 Deployment of VBX Stent and Ventricular Septal Defect Occluder (Modified Tootsie Roll Technique)

Video 6 Postclosure Aortogram

Video 7 TEE Image After Closure of Fistula With Occluder Device
